# Case Report: Catheter-Directed Endovascular Thrombolysis for Refractory Cerebral Venous Sinus Thrombosis in a Patient With Behçet Disease

**DOI:** 10.3389/fneur.2021.642088

**Published:** 2021-02-23

**Authors:** Yongfeng Xu, Minjian Qiu

**Affiliations:** Department of Neurology, The Second Affiliated Hospital of Zhejiang University School of Medicine, Hangzhou, China

**Keywords:** Behçet disease, cerebral venous sinus thrombosis, catheter-directed endovascular thrombolysis, intracranial hypertension, anticoagulant therapy

## Abstract

**Background:** Behçet disease (BD) is an autoimmune and vasculitic disorder. BD affects the veins more frequently than the arteries. The cerebral venous sinus thrombosis (CVST) was reported to occur in about 20% of neuro-BD. Huge dilemma exists in the treatment of CVST with BD, some of which were refractory to the standard therapy.

**Case Presentation:** Here, we report a BD case with refractory intracranial hypertension caused by CVST which is successfully treated with catheter-directed endovascular thrombolysis. Before endovascular thrombolysis, intravenous pulse methylprednisolone combined with anticoagulant therapy was used, and resulted in limited effects.

**Conclusion:** We conclude that catheter-directed thrombolysis may be considered for refractory CVST with BD, provided that coexistent pulmonary and cerebral artery aneurysms are ruled out.

## Introduction

Behçet disease (BD) is characterized by recurrent oral aphthous ulcers and other systemic manifestations. BD is believed to be an autoimmune and vasculitic disorder. BD affects the veins more frequently than the arteries (1). The vascular inflammation causes a significant venous thrombotic tendency. The lower extremity venous thrombosis is most frequent, but other veins, including cerebral venous sinus, can also be affected. The cerebral venous sinus thrombosis (CVST) was reported to occur in about 20% of neuro-BD (2). Huge dilemma exists in the treatment of CVST with BD. Here, we report a BD case with refractory intracranial hypertension caused by CVST which is successfully treated with catheter-directed endovascular thrombolysis. Before endovascular thrombolysis, intravenous pulse methylprednisolone combined with anticoagulant therapy was used, and resulted in limited effects.

## Case Presentation

**Figure 1:**
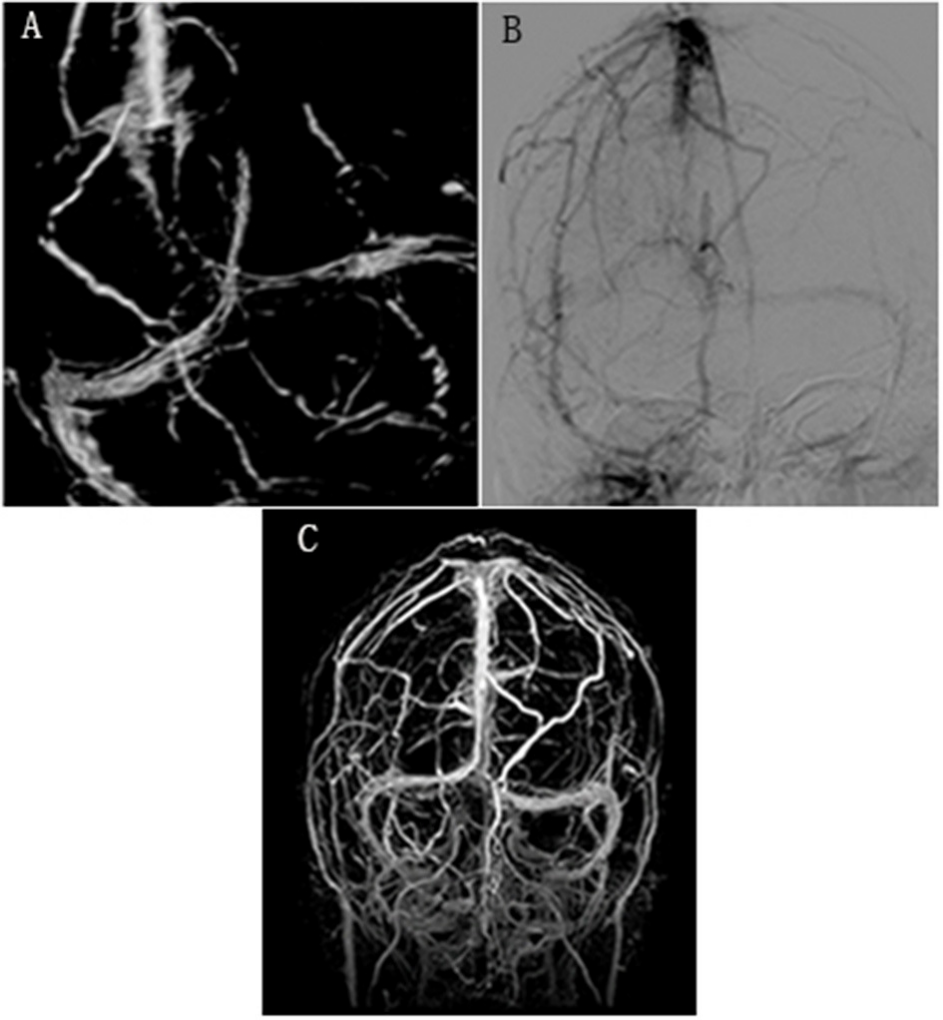
**(A)** Brain magnetic resonance venography (MRV) showed that venous thrombosis of bilateral transverse sinus, sigmoid sinus, and superior sagittal sinus in June 14, 2016. **(B)** Digital subtraction angiography (DSA) showed that venous thrombosis of right transverse sinus, sigmoid sinus, and superior sagittal sinus before catheter-directed endovascular thrombolysis in September 1, 2016. **(C)** Brain MRV showed that venous thrombosis of superior sagittal sinus, transverse sinus, and sigmoid sinus was improved significantly after catheter-directed endovascular thrombolysis.

A 34-year-old male patient was admitted with a 1-month history of headache in June 14, 2016. The patient has no fever and no other symptoms. In 2012, he had recurrent oral aphthous ulcers, epididymitis, uveitis, papulopustular lesions, and arthritis, then he was diagnosed as BD according to the International Criteria for Behçet Disease (ICBD). Although he used prednisolone (8 mg per day) and thalidomide (50 mg per day) regularly, he had recurrent epididymitis. Other past medical history and family history were unremarkable. Physical examination on admission showed papulopustular lesions on the back, and no abnormality was found in the nervous system. Brain magnetic resonance imaging (MRI) showed no abnormality. Brain magnetic resonance venography (MRV) showed that venous thrombosis of bilateral transverse sinus, sigmoid sinus, and superior sagittal sinus (Figure 1A). Lumbar puncture showed that the pressure was elevated significantly (>400 mmH_2_O). Cerebrospinal fluid (CSF) analysis showed normal cellularity (4 cells/μl), protein (43 mg/dl), glucose (3.98 mmol/L), and chloride concentrations (118.9 mmol/L). Ink staining was negative. CSF culture was negative. CVST was considered. Coagulation profile was normal. Protein C, Protein S, and antithrombin III were normal. C-reactive protein (CRP) and erythrocyte sedimentation rate (ESR) levels were 89 mg/L (normal 0–5 mg/L) and 31 mm/h (normal 0–20 mm/h), respectively. Antinuclear antibody (ANA), antiphospholipid antibodies and antineutrophil cytoplasmic antibodies were negative. TPPA antibody of syphilis and HIV antibody were negative. CVST related to the BD was considered. Ultrasonography revealed epididymal inflammatory lesions. Ultrasonography of lower extremity artery and vein, CT angiography of pulmonary artery, and cranial MRA were all normal. Intravenous pulse methylprednisolone (1,000 mg per day for 3 days, then 500 mg per day for 5 days, then prednisone 60 mg per day) combined with low molecular weight heparin (LMWH) was added. Mannitol and glycerol fructose were used to reduce intracranial pressure when necessary. The patient's headache improved slightly. In June 22, 2016, brain MRV showed that the CVST was not improved (not shown). Lumbar puncture was repeated and showed that the pressure was elevated significantly (360 mmH_2_O). Warfarin and prednisone (60 mg per day) were maintained. Azathioprine (2 mg/kg) was added. Then the patient was transferred to another hospital for the treatment. In August 16, 2016, the patient was readmitted to our department because of severe headache. Brain MRV showed that the venous thrombosis was similar to that of June 22, 2016. Lumbar puncture was repeated and showed that the intracranial pressure was still elevated significantly (>400 mmH_2_O). The benefits and risks of catheter-directed endovascular thrombolysis for the refractory CVST was discussed with the patient. In September 1, 2016, brain digital subtraction angiography (DSA) (Figure 1B) showed that venous thrombosis of right transverse sinus, sigmoid sinus and superior sagittal sinus. Then catheter-directed endovascular thrombolysis was performed. Through the right femoral vein, a 6F 90 cm guiding catheter (Cook Corporation) was introduced into the right internal jugular vein. Then, under the guidance of a guidewire, a microcatheter was placed into the superior sagittal sinus. Urokinase (42,000 U/h, total 1,000,000 U per day) was used and administered continuously into the sinus by microcatheter. Heparin was also administered continuously through the guiding catheter. After 5 days of thrombolysis, the headache was relieved, and brain MRV was repeated and showed that venous thrombosis of superior sagittal sinus, bilateral transverse sinus, and sigmoid sinus was improved significantly (Figure 1C). The patient was discharged. Warfarin, corticosteroids, and azathioprine were maintained. The dose of the corticosteroids was reduced gradually and low dose corticosteroids (10 mg per day) was maintained. Brain MRV was repeated in August 2017, April 2019, and June 2020, respectively, and showed that there was no new sinus thrombosis (not shown).

## Discussion

Vasculitis which can involve the veins and arteries is the main pathological process in BD. The vasculitis in BD affects the veins more frequently than the arteries, and causes a significant venous thrombotic tendency. It is speculated that inflammation causes the dysfunction of endothelial cells, which leads to inhibition of fibrinolysis and natural anticoagulant pathways, and leads to activation of platelet (1).

Huge dilemma exists in the treatment of CVST with BD. The European League Against Rheumatism (EULAR) agreed that high dose glucocorticoids should be used for acute CVST. No data shows the benefit of adding immunosuppressants in the acute CVST, and immunosuppressants may also be unnecessary for the relapses of the CVST with BD. Anticoagulants may be added, but only for a short time (2). It is reported recently that a patient with CVST related to BD came with headaches and vision changes that was resolved after 5 sessions of plasmapheresis along with steroids and anticoagulation, which provides another alternative option of treatment (3). In acute deep venous thrombosis other than CVST, glucocorticoids and immunosuppressants such as cyclophosphamide are recommended, and anticoagulants may be added provided that coexistent pulmonary artery aneurysms are ruled out (2).

Systemic anticoagulation (LWMH or heparin) is the mainstay treatment of CVST. However, about 9–13% of the CVST patients have poor outcome despite the anticoagulation therapy (4). The factors associated with severe CVST include coma, thrombosis of the deep cerebral venous system, central nervous system infection, and cancer, etc (5). The urgent relief of the cerebral venous hypertension is very important for the patients who deteriorate despite anticoagulation therapy. While the endovascular intervention is not the first line therapy, it has been used as salvage treatment for severe CVST which is refractory to anticoagulation therapy (4). In CVST with BD, it is reported that although 85.7% (18 patients) of the patients achieved remission with the treatment of glucocorticoid, immunosuppressant therapy and anticoagulants, 14.3% (3 patients) required systemic thrombolytic treatment (6). In a systematic review of CVST with BD, sequelae were present in 20% of the patients. Optic nerve atrophy and blindness were the predominant sequel (7). It is reported that thrombolysis therapy was useful in deep venous thrombosis (8), pericarditis (9) with BD, and thrombolysis therapy was also tried in Budd-Chiari syndrome with BD (10). In this case, we also report a refractory CVST with BD was treated successfully with catheter-directed endovascular thrombolysis. Before thrombolysis therapy, we excluded the pulmonary and cerebral aneurysms. There was no major hemorrhage in our case.

We conclude that catheter-directed thrombolysis may be considered for refractory CVST with BD, provided that coexistent pulmonary and cerebral artery aneurysms are ruled out.

## Data Availability Statement

The original contributions presented in the study are included in the article/supplementary material, further inquiries can be directed to the corresponding author/s.

## Ethics Statement

The studies involving human participants were reviewed and approved by Second affiliated hospital, school of medicine, zhejiang university. The patients/participants provided their written informed consent to participate in this study. Written informed consent was obtained from the individual(s) for the publication of any potentially identifiable images or data included in this article.

## Author Contributions

YX and MQ provided the idea. YX wrote the manuscript. MQ revised the manuscript and did the proofreading.

## Conflict of Interest

The authors declare that the research was conducted in the absence of any commercial or financial relationships that could be construed as a potential conflict of interest.
